# Retraction: Corrigendum: Identification of cerebral metal ion imbalance in the brain of ageing *Octodon degus*

**DOI:** 10.3389/fnagi.2026.1822870

**Published:** 2026-03-11

**Authors:** Frontiers Editorial Office

The journal retracts the May 15 2017 article cited above.

Following publication, concerns were raised regarding the integrity of the images in the published figures. An investigation was conducted in accordance with Frontiers' policies. Given the concerns about the validity of the data and the lack of original raw images, we no longer have confidence in the findings presented in the article.

The retraction was approved by the Chief Executive Editor at Frontiers. The authors were informed of the retraction and had an opportunity to respond. This communication has been recorded by the publisher.

